# Staff and patient experiences of decision-making about continuous observation in psychiatric hospitals

**DOI:** 10.1007/s00127-017-1338-4

**Published:** 2017-02-04

**Authors:** Kirsten Barnicot, Bryony Insua-Summerhayes, Emily Plummer, Alice Hart, Chris Barker, Stefan Priebe

**Affiliations:** 1 Department of Medicine, Centre for Psychiatry, Imperial College London, Commonwealth Building, Du Cane Road, London, W12 0NN UK; 2 Department of Medicine, Clinical, Educational and Health Psychology, University College London, 1-19 Torrington Place, London, WC1E 7HB UK; 3 Department of Neuroscience, Physiology and Pharmacology, University College London, Gower Street, London, WC1E 6BT UK; 4 Unit for Social and Community Psychiatry, Department of Medicine, Queen Mary University of London, Glen Road, London, E13 8SP UK

**Keywords:** Inpatients, Psychiatric hospitals, Risk management, Patient rights, Qualitative

## Abstract

**Purpose:**

Continuous observation of psychiatric inpatients aims to protect those who pose an acute risk of harm to self or others, but involves intrusive privacy restrictions. Initiating, conducting and ending continuous observation requires complex decision-making about keeping patients safe whilst protecting their privacy. There is little published guidance about how to balance privacy and safety concerns, and how staff and patients negotiate this in practice is unknown. To inform best practice, the present study, therefore, aimed to understand how staff and patients experience negotiating the balance between privacy and safety during decision-making about continuous observation.

**Methods:**

Thematic analysis of qualitative interviews with thirty-one inpatient psychiatric staff and twenty-eight inpatients.

**Results:**

Most patients struggled with the lack of privacy but valued feeling safe during continuous observation. Staff and patients linked good decision-making to using continuous observation for short periods and taking positive risks, understanding and collaborating with the patient, and working together as a supportive staff team. Poor decision-making was linked to insufficient consideration of observation’s iatrogenic potential, insufficient collaboration with patients, and the stressful impact on staff of conducting observations and managing risk.

**Conclusions:**

Best practice in decision-making about continuous observation may be facilitated by making decisions in collaboration with patients, and by staff supporting each-other in positive risk-taking. To achieve truly patient-centred decision-making, decisions about observation should not be influenced by staff’s own stress levels. To address the negative impact of staff stress on decision-making, it may be helpful to improve staff training, education and support structures.

**Electronic supplementary material:**

The online version of this article (doi:10.1007/s00127-017-1338-4) contains supplementary material, which is available to authorized users.

## Introduction

During their stay in hospital, 13–16% of psychiatric inpatients will be placed on continuous observation, remaining within eyesight of a member of staff at all times [1–3]. This has significant resourcing implications, accounting for up to 20% of the total USA nursing budget, and costing the UK NHS £35 million per annum [4, 5]. Whilst commonly prescribed to protect patients who pose an acute risk of harm to self or others, the concomitant invasion of privacy can cause patients distress and undermine their relationships with staff [6–14]. Yet it also affords patients and staff a unique period of one-to-one time together which may not ordinarily be available in the busy ward environment, and can, therefore, be an important opportunity to build trusting therapeutic relationships between patients and staff [7, 8, 12–14]. Deciding when to increase or decrease patients’ level of observation, and their level of privacy during it, involves complex decision-making about how to optimally balance safety versus privacy. Some national guidelines recognise this tension, with the UK National Institute for Health and Care Excellence (NICE) guideline on the management of violence recommending “Use the least intrusive level of observation necessary, balancing the service user’s safety, dignity and privacy with the need to maintain the safety of those around them” [3]. However, NICE offers little specific guidance on how to decide this balance, and in practice nursing observations continue to be governed by local-level policies. These vary widely [1], with some making minimal reference to privacy concerns and offering entirely risk-focussed guidance for deciding observation levels [15, 16]. To inform best practice in decision-making around continuous observation, it is, therefore of vital importance to understand how staff and patients negotiate the balance between safety and privacy in practice. Qualitative methodology can yield insights into real-world processes that cannot be captured by numerical data [17]. Existing qualitative studies on continuous observation have not explored how staff and patients negotiate the balance between safety and privacy and have employed small sample sizes. Moreover, few studies have integrated staff and patient experiences, nor interviewed patients with heterogenous risk presentations, nor staff across multiple levels of seniority [6–14].

## Aims of the study

The present study, therefore, aimed to address the following question:

How do staff and patients experience decision-making about balancing safety and privacy when initiating, conducting and ending continuous observation?

To address this question, the study aimed to triangulate the perspectives of patients across a variety of diagnostic and risk presentations with the experiences of staff across a range of professional backgrounds and levels of seniority.

## Materials and methods

### Design

Qualitative interviews with psychiatric inpatients and staff about their experiences of continuous observation.

### Inclusion criteria

Patients were included if they:

1. Had been on continuous observation within the past year and could recall their experiences
2. Had capacity to consent to an interview

Staff were included if they:

1. Had conducted or managed continuous observation of a patient within the past year

### Recruitment and sampling

Participants were recruited from adult acute wards in two psychiatric hospitals in two inner London NHS Trusts, situated in two of the most deprived and ethnically diverse local authorities in the United Kingdom [18, 19]. Staff identified eligible patient interviewees from their case-list. Ward managers identified eligible staff.

Sampling frameworks were used to facilitate maximum variation sampling [20], by guiding purposive recruitment of patients and staff with different combinations of characteristics that could influence experiences of continuous observation. The patient framework encompassed diagnosis (psychotic disorder, non-psychotic disorder), reason for observation (harm to self, harm to others) and length of time under observation (≤7 days, >7 days). The staff framework encompassed gender (male, female) and professional role (unqualified nursing staff, qualified nursing staff, senior management (ward managers, modern matrons, consultant psychiatrists or psychologists)).

Recruitment was terminated when (1) at least one individual had been interviewed for each combination of characteristics specified in the sampling frameworks, and (2) study authors judged that additional interviews were not contributing new ideas to the analysis (‘data saturation’) [21].

### Data collection

The study was approved by the Surrey and South East Coast NHS Research Ethics Committee (13/LO/0531). All participants gave written informed consent prior to participating.

Qualitative interviews explored participants’ experiences of privacy, safety and decision-making during continuous observation and were based on a semi-structured interview schedule, developed with input from a patient with past experience of continuous observation, with some key questions followed by flexibly worded suggested probes for further exploration. The interviews were conducted by KB, BIS, EP or AH in a private room on the ward or an adjacent building, lasted 10–90 min, and were audio-recorded.

Participating patients gave informed consent for researchers to review their electronic notes to confirm their age, diagnosis, and the length of and reason for observation.

### Interview analysis

The authors took a critical realist approach, viewing participants’ accounts as grounded in reality, but acknowledging the influence of subjectivity and the social context on data collection and analysis [22]. To ensure credibility of study findings, a team-based approach was used [23], whereby coding was led by KB and BSH, regularly reviewed and critiqued in-depth by EP and AH, and findings discussed by all authors at regular intervals. Analysis and data collection occurred concurrently using an iterative approach so that early analysis informed conduct of subsequent interviews [21]. Using thematic analysis [24], transcripts were coded using NVivo software [25], codes were sorted into preliminary themes, and then repeatedly reviewed and refined to maximise internal homogeneity and external heterogeneity, until all authors agreed that the themes and sub-themes accurately reflected the overall ‘narrative’ of the data.

### Quality assurance and reflexivity

The authors adhered to Elliott and colleagues’ guidelines for qualitative research [26], and the consolidated criteria for reporting qualitative research (CORE-Q) [27]. All authors have a background in academic psychology or psychiatry, none have personally conducted continuous observation, and some have themselves experienced continuous observation during past experiences of using mental health services. Whilst aiming to approach data collection and analysis without a priori hypotheses, the authors engaged in ‘mindful inquiry’ [28], by noticing, accepting and transcending the influence of their own beliefs, knowledge and experiences, facilitated by the use of note-taking and memos.

## Results

**Fig. 1:**
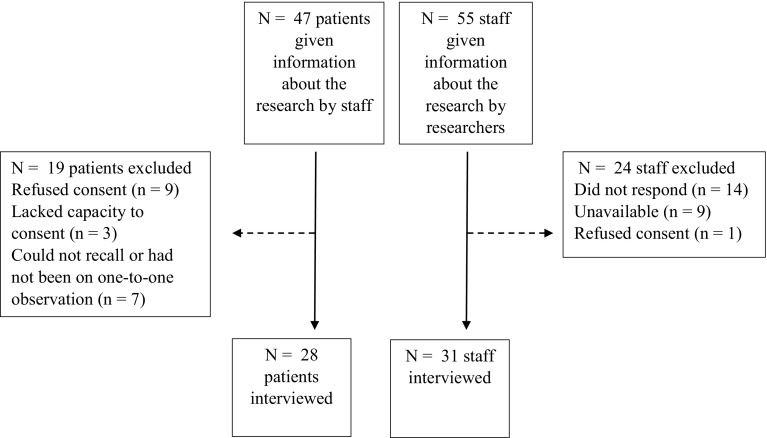
Participant recruitment

Twenty-eight inpatients and thirty-one staff participated. Participant recruitment is shown in Fig. 1, whilst Table 1 describes their sociodemographic, clinical and professional characteristics.

**Table 1:** Characteristics of interviewed inpatients and staff

Patients (*N* = 28)	
Gender	
Male	15 (54%)
Female	13 (46%)
Ethnicity *N* (%)	
White	14 (50%)
Asian	7 (25%)
Black	6 (21%)
Mixed race	1 (4%)
Age (years)	
Range	18 to 66
Mean (sd)	37 (15)
Primary diagnosis *N* (%)	
Personality disorder	10 (36%)
Bipolar disorder	9 (32%)
Schizophrenia or schizoaffective disorder	5 (18%)
Major depressive disorder	4 (14%)
Reason for observation *N* (%)	
Risk to self	18 (64%)
Risk to others	6 (21%)
Risk to self and risk to others	4 (15%)
Length of time on observation *N* (%)	
≤7 days	12 (43%)
>7 days	16 (57%)
Staff (*N* = 31)	
Gender *N* (%)	
Male	15 (48%)
Female	16 (52%)
Ethnicity *N* (%)	
White	17 (55%)
Asian	3 (10%)
Black	11 (35%)
Mixed race	0 (0%)
Years worked in mental health	
Range	1 to 25
Mean (sd)	7 (7)
Job role *N* (%)	
Unqualified nursing staff	12 (39%)
Qualified nursing staff	9 (29%)
Clinical team leader	2 (6%)
Ward manager	3 (10%)
Modern matron	1 (3%)
Consultant psychiatrist	3 (10%)
Consultant clinical psychologist	1 (3%)

**Fig. 2:**
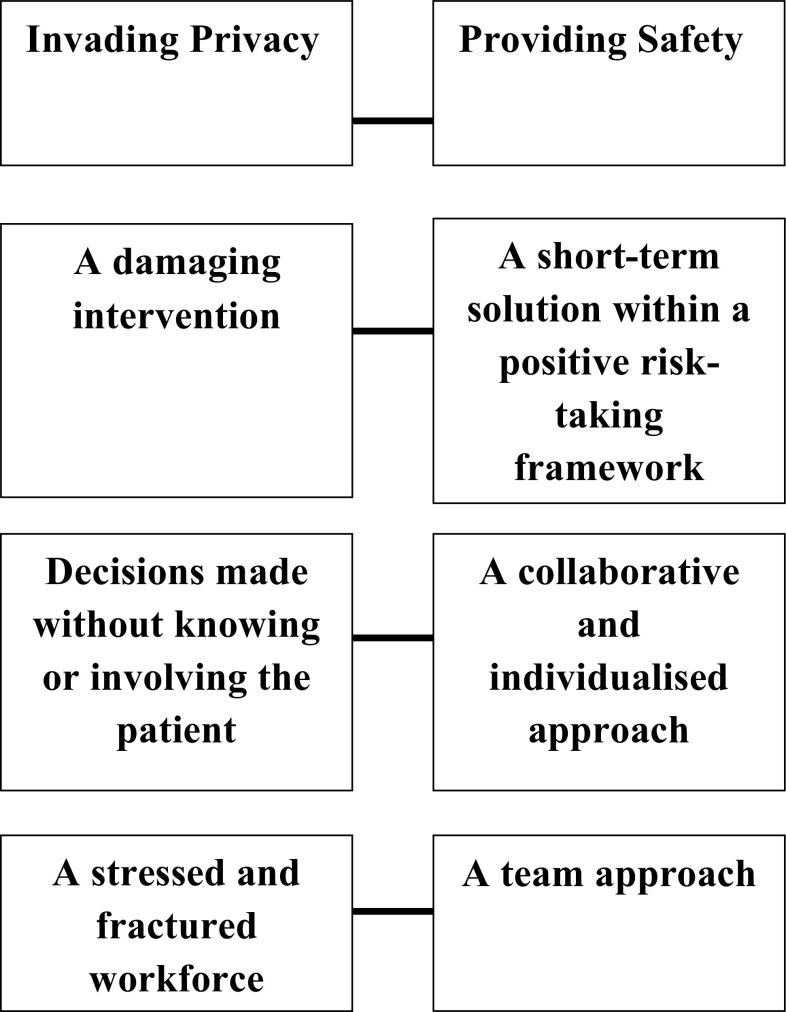
Overview of the thematic framework

Through thematic analysis of interviews with patients and staff, four themes, with two sub-themes each, were derived (Fig. 2).

Theme 1 encapsulates the inherent conflict between maintaining both privacy and safety during observation. In Themes 2–4, one sub-theme characterises factors that make it difficult to balance privacy and safety, and the other characterises factors that help in negotiating this conflict. Numbers endorsing each sub-theme are reported, although frequency should not be taken as the sole indicator of salience [29, 30].

Quotes from patient interviews are prefaced ‘P’ and quotes from staff ‘S’. Additional supporting quotes from participants are available in online Supporting Information Table S1. Patients and staff tended to use the informal term “one-to-one” to refer to continuous observation and this is reflected in the quotes.

### Theme 1 The conflict between privacy and safety

#### Sub-theme 1.1 Invading privacy

Almost all patients (23/28) and staff (28/31) recognised that continuous observation constituted a significant invasion of patients’ privacy. Patients described the lack of privacy during continuous observation as very intrusive. Staff were also very aware that many patients find this difficult.

##### *S18:*

*“I do understand, sometimes we invade their space, sitting there, watching them… But we have to do it, because, if they are harming themselves we need to watch them all the time”*.

Patients found the lack of privacy particularly difficult when showering, using the toilet, or trying to sleep. At times, the intrusiveness of the intervention made them feel powerless, degraded, or punished.

##### *P01:*

*“You’re constantly being watched, your every movement. You lose your space and it feels like you’re being invaded and they’re in control of you”*.

#### Sub-theme 1.2 Providing safety

The majority of patients (20/28) and all staff (31/31) recognised that continuous observation could be valuable for preventing patients harming themselves or others.

##### *S16:*

*“One-to-one observation is very important, we can’t take one-to-one out because then patients will end up dying”*.

Patients often gained a sense of safety and comfort from the constant presence of staff, particularly if they felt frightened by their urges to hurt themselves or others, and were, therefore, willing to accept the invasion of their privacy as a necessary trade-off.

##### *P13: *

*“I felt safe because I couldn’t trust myself, and I felt they were keeping me safe …it saved me from myself”*.

Others were more conflicted about whether they wanted to be kept safe, either moving from anger to grudging acceptance, or fighting to be allowed to hurt themselves but feeling abandoned once staff reduced their level of observation.

##### *P28:*

*“There was a large part of me that didn’t want to be kept safe…. I was adamant that I didn’t want to go on one to one… [but] in some ways almost when they did say ‘Okay’ I thought ‘Oh they’ve given up on me now’”* .

### Theme 2 A damaging intervention versus a short-term solution within a positive risk-taking framework

#### Sub-theme 2.1 A damaging intervention

The majority of staff (26/ 31) and half of the interviewed patients (14/28) highlighted that, in some cases, being placed on continuous observation could actually increase rather than decrease patients’ risk. First, the intrusion of privacy and restriction of liberty could increase patients’ anger and paranoia towards staff, leading to increased levels of aggression and undermining staff–patient relationships.

##### *S28:*

*“If patients feel like being restricted or being controlled… you can’t build any sense of trust…it could be a detriment to their recovery process”*.

Second, the feeling of entrapment could increase their urges to hurt themselves.

##### *P05:*

*“I hate being stared at. It makes me agitated.....It made me do it [self-harm] more.... because of the amount of pressure they put on me”*.

Staff raised the possibility that placing patients on continuous observation could reinforce risk behaviours on an unconscious level, by implicitly rewarding their behaviour with increased time and attention from staff, and could increase patients’ dependency on staff.

##### *S12:*

*“It might actually end up making the situation worse by reducing the patient’s sense of self-efficacy and their own skills in managing the situation…. you can inadvertently reinforce some of the behaviours”*

#### Sub-theme 2.2 A short-term solution within a positive risk-taking framework

About half of the interviewed staff (15/31) emphasised that continuous observation was most effective when used as a short-term solution for only the most severe levels of risk, whereas lower risk patients should be managed using less intrusive methods such as intermittent observation or debriefing. Where continuous observation proved to be iatrogenic, or where decreases in risk occurred, staff advocated positive-risk taking, such as allowing additional privacy or reducing the level of observation.

##### *S12:*

*“It’s a useful acute risk management tool....If they’re self-harming in a very serious way that’s likely to lead to permanent damage, as a short-term measure, in an acute situation, you might use one to one... if it’s something that’s a long-term problem and you need to be looking at long-term solutions....Because at some point you have to have positive risk taking”*.

They stressed that continuous observation should not be used as a knee-jerk reaction to risk, and that observation alone could not solve patients’ underlying long-term difficulties and should be used in tandem with therapeutic input.

##### *S02:*

*“Sometimes people are put on one-to-one as a reaction to something that’s happened.... One to one isn’t really gonna solve anything. Sometimes it might just be better to have a debrief. Talk through what’s happened and why it’s happened. And then, alright, let’s just try to continue to move forward”*.

### Theme 3 Decisions made without the patient versus a collaborative and individualised approach

#### Theme 3.1 Decisions made without knowing or involving the patient

The majority of patients (22/28) and a third of staff (10/31) emphasised that observation was more likely to be unhelpful when decisions were made without adequate knowledge or involvement of the patient. Patients felt the intervention was particularly unhelpful when they were not informed about the nature and rationale for decisions about observation, or when their own opinions about their needs and their level of risk were disregarded, so that they were placed or maintained on observation unnecessarily, or unfairly restricted during it.

##### *P07:*

*“I knew that I wasn’t a danger to myself ... I was in a safe environment.... I didn’t need to be watched... I didn’t see the sense in it”*.

Staff acknowledged that they were more likely to err on the side of caution for less well-known patients, and explained that finding time to truly get to know patients could be difficult in busy and demanding ward environments.

##### *S25:*

*“Sometimes a patient comes in and we will tell an HCA go and do a one-to-one... yet we’ve not even had the opportunity to read up on the risks. It’s important that a patient comes in and we get to know the risk before we say ‘go do the one-to-one’”*.

Staff also cautioned that some patients could be very unpredictable, whilst others could deliberately try to hide their level of risk through their desperation to find a way of carrying out their plans for self-harm.

#### Theme 3.2 A collaborative and individualised approach

Almost all staff (30/31) and about half of the patients (15/28) spoke about the importance of formulating a thorough assessment of the individual patient’s presentation to inform decisions about starting and ending observation, and to allow staff to flexibly judge how much privacy to give individual patients during observation. Developing a thorough understanding of the patient was greatly facilitated if staff were able to build rapport—and staff emphasised a vital starting point was to ensure that the patient understood why they had been placed on observation and what it would involve, and to acknowledge that the lack of privacy could be difficult and frustrating.

##### *S16:*

*“It’s all about communicating, ‘This is why I’m sitting with you, for your safety; I’m here to help you. Even if you don’t like it, just trust on that I’m here to help you. You are safe’”*.

The development of mutual trust between patient and staff could then allow staff to collaborate with the patient to take more positive risks, such as agreeing with the patient that they could use the bathroom in privacy but that staff would knock frequently to check on their welfare.

##### *P22:*

*“After a while they started to trust me- like ‘I’m going to the lavatory, nurse, I’ll be about 5 minutes’ they’ll let me go. And if I’m not back in 5 min, then they’d come and look for me”*.

##### *S30:*

*“If you’re beginning to know a bit more about who they are, you might feel able to take greater therapeutic risks, in the hope of encouraging them to take responsibility”*.

### Theme 4 A stressed and fractured workforce versus a team approach

#### Sub-theme 4.1 A stressed and fractured workforce

Almost all staff (28/31) raised concerns that the stressful nature of observation and risk management could disproportionately influence decision-making and lead to inconsistent implementation of observation between different staff members. Some patients (9/28) were also uncomfortably aware of the effect of staff emotions and inconsistency on decisions about their observation. Staff explained that decision-making around continuous observation could be affected by high levels of anxiety about preventing patients harming themselves or others, linked to worries about being blamed, which in turn could lead to enforcing greater privacy restrictions or maintaining patients on continuous observation unnecessarily.

##### *S21:*

*“We’ve got to cover our backs at the end of the day …. if she does something that she shouldn’t really do, then we are the ones in trouble, and sometimes you do feel that that does influence your decisions”*.

##### *S28:*

*“What restricts privacy is the fact that staff are anxious that they’re culpable for whatever happens for that one hour, [which] makes staff adopt a black and white approach, a restrictive approach”*.

Equally, both staff and patients highlighted that continuous observation could not always prevent patients harming themselves or others. Staff emphasised that the requirement for constant vigilance during continuous observation could be both emotionally and physically draining, and both staff and patients highlighted that even momentary lapses in vigilance could enable patients to hurt themselves in unanticipated ways such as scalding themselves with boiling water from a tea urn or strangling themselves with their bed-sheets. Lapses in vigilance were particularly linked to staff growing tired, letting their guard down at night, being left to conduct observation for long periods without being replaced due to staff shortages, or patients being allowed to use the bathroom on their own if a same-sex member of staff could not be located.

##### *P28:*

*“There was one time where I managed to ligature in the bathroom whilst I had a male member of staff, and it took them about ten minutes to come and find me because they had to wait and get a female member of staff to come and break into the bathroom”*.

Staff also reported being particularly anxious when observing patients of the opposite sex, due to past allegations of inappropriate staff behaviour, and therefore, took extra precautions such as observing from a greater distance or leaving the door open.

Experiences of verbal or physical aggression from patients could also be very stressful and made staff reluctant to carry out the observation.

##### *S03:*

*“If someone was aggressive, being on one-to-one is not safe for staff. It gets quite hard because you just think ‘Oh god, I don’t actually want to go into their room, I want to stay right outside their room’”*.

Staff also explained that, given staff shortages and low staff to patient ratios, allocating one member of staff to just one patient could add to staff stress by reducing their capacity to address the needs of other patients. This also added to financial pressures if additional bank or agency staff needed to be brought in to boost staff numbers, which in turn could increase pressures on ward managers to reduce patients’ level of observation. Staff reported that differing opinions within the team about the necessity of continuous observation could lead to ‘splitting’ or inconsistency in the degree of privacy granted during observation. Patients picked up on this splitting and inconsistency, finding it uncomfortable and frustrating.

##### *P21:*

*“Slap-dash approach, leaving me alone, wouldn’t bother to do the one-to-one half the time...... It really upset me because I felt really judged... the conflict between nurses made me feel worse”*.

##### *S31:*

*“The team was quite split at the time, it was one side that just did not want to take any risks and keep her on one-to-one, and then it was the other side who felt ‘we need to take a risk, we need to take her off one-to-one or she will continue to do this’”*.

#### Sub-theme 4.2 A team approach

Almost all staff (27/31) emphasised that collaboration as a team was crucial in supporting them to make patient-centred decisions and helping them cope with the stresses of continuous observation. They highlighted that decisions about patients’ observation level should be made as a team, with all staff taking a unified stance to prevent splitting. Additionally, information gained by one member of staff during observation should be handed over to the rest of the team, to help the whole team get a better understanding of the patient.

##### *S14:*

*“If they like you then they’ll tell you more than the other staff, so whosoever goes there gets more information can pass it on to the team and the team can come up with a plan….to work towards those issues”*.

Both practical and emotional support from colleagues were perceived to be very important. Practical support included maintaining an adequate level of staffing on the ward so that other staff were available to take over rather than leaving one person to conduct observation for more than an hour, could support the observing member of staff should an emergency occur, and were still able to adequately cater for the needs of other patients on the ward. The opportunity to share and reflect on difficult experiences with colleagues, both informally and through formal supervision and reflective practice, was also highly valued.

##### *S09:*

*“We usually try to swap regularly, to make sure that the person who is on one-to-one will have time off and can at least have a cup of tea. Then reflection - usually the staff who is able to make conversation with the person on one-to-one is quite keen to share everything with the team, so in a full discussion with the team you reduce your stress”*.

##### *S31:*

*“I do encourage staff to make decisions for themselves and for them to have a good reason behind the decision making that they have. There’s a very clear supervision structure as well, so that they can discuss their decisions. And we have reflection with the psychotherapist where we would bring up decisions about one-to-one, discuss it as a team and come to—if not complete agreement—at least a centralised hymn sheet that we would work from”*.

## Discussion

Through thematic analysis of interviews with inpatients and staff, we derived four bipolar themes characterising factors that make it difficult to balance privacy and safety, and factors that help in negotiating this conflict. The main findings were that both staff and patients were keenly aware of the conflict between privacy and safety during continuous observation. Whilst many patients reported finding the lack of privacy to be intrusive and disempowering, many were also grateful for the feeling of safety it provided, although others were more conflicted about whether they wanted to be protected in this way. Three key factors were reported by both patients and staff to contribute to poor decision-making around balancing privacy and safety: insufficient consideration of the iatrogenic potential of observation, insufficient understanding of or collaboration with patients, and staff stress linked to anxiety about risk in tandem with the stressful demands of observation. Three key factors were reported to contribute to good decision-making around balancing privacy and safety: using continuous observation as a short-term solution within a positive risk-taking framework, understanding and collaborating with the patient as an individual, and working together as a mutually supportive staff team.

Previous work has indicated that conducting continuous observation has staff resourcing implications and is stressful, linked to increases in staff sickness, and that disagreements between staff regarding the necessity of observation are common [1, 31]. Lapses in vigilance during observation, reported in our study to be linked to staff stress, have been linked to patient deaths [32]. Conversely, our finding that continuous observation can be iatrogenic, with the potential to increase risk and slow recovery, has not previously been shown to our knowledge. In our study, weighing up the potential benefits versus negative consequences of continuous observation was described as a complex task requiring a thorough knowledge of the individual patient’s current and past presentation and responses to observation. The importance of communication and collaboration with patients, and of a supportive and cohesive staff team, has been emphasised in previous studies interviewing nurses about continuous observation [8, 10, 13, 14]. More widely, UK Department of Health guidance on risk management emphasises the value of taking positive risks, whereby staff should involve patients in decisions and should be willing to take decisions involving an element of risk if this is outweighed by potential benefits [33].

## Strengths and limitations

This was to our knowledge the largest study to date triangulating patient and nurse experiences of continuous observation. The potential to generalise study findings was increased by recruitment of patients with a range of diagnoses and risk profiles, and a range of staff roles at different levels of seniority, across two NHS Trusts. Some study authors had themselves experienced being on continuous observation, ensuring that patient experiences remained central to the analysis rather than being dominated by academic or clinical perspectives.

A limitation was that some patients found it difficult to recall their experiences of observation, particularly if it had occurred weeks previously or they had been heavily medicated. Both participating hospitals were in inner city London boroughs—the findings may not be generalisable to hospitals elsewhere.

### Implications for clinical practice and research

Based on the themes generated from interviews with staff and patients in the present study, Table 2 describes lessons learnt for best practice in decision-making around continuous observation. Whilst we cannot determine based on these subjective qualitative accounts whether these recommendations would improve patient outcomes, they could be combined with those made in existing policy and research [3, 6, 10, 33, 34], to develop and test an intervention to support staff in collaborating with each other and with patients to implement patient-centred and positive risk-taking decisions about continuous observation.

Observation policies are largely locally determined and vary widely [1], with many focussed only on risk when outlining how to decide patients’ level of observation and privacy [15, 16]. By contrast, in the present study both patients and staff consistently highlighted that decisions about continuous observation are most helpful for patients’ overall recovery when they are made in collaboration with patients and taking into account any potential negative effects of observation. Staff accounts also emphasise the importance of supporting each-other to make decisions as a team and to take positive risks. Conversely, the accounts of the negative impact of staff stress on decision-making are concerning, and reflect reports of wider problems in psychiatry of understaffing [35–37] and a culture of blame [38]. If understaffing and ward overcrowding means that staff conducting observations are not regularly replaced, or are drawn into incidents elsewhere on the ward, lapses in vigilance may occur and risk to patients may increase. Conversely, if a culture of risk aversion linked to a fear of blame is allowed to predominate, then patients may be unnecessarily kept on continuous observation, staff-patient relationships may suffer, and the patient’s overall recovery may be hindered. The ability of staff to engage in positive risk-taking is very much determined by the willingness of management to encourage staff in this, and staff should feel confident that management will continue to support their decision to take a positive risk even in the rare case that a serious escalation in risk behaviour subsequently occurs. Addressing these problems may require a top-down cultural shift in inpatient psychiatry [39].

**Table 2:** Lessons learnt for best clinical practice in decision-making about continuous observation

Findings	Considerations raised by patients and staff
Interviewees felt continuous observation was best used as a short-term intervention within a positive-risk-taking framework	Ensure that only patients with severe levels of risk are placed on continuous observation Frequently re-evaluate risk during observation Be open to taking positive risks during or as an alternative to continuous observation Keep the duration of continuous observation to a minimum
Interviewees felt continuous observation had the potential to be iatrogenic	Consider the potential negative effects of continuous observation for the individual patient, including distress caused by privacy restrictions, reinforcement of risk-taking behaviour, reduced self-efficacy and negative relationships with staff
Interviewees felt good decision-making required a thorough knowledge of the individual patient	Avoid knee-jerk or blanket reactions to risk behaviour Thoroughly evaluate all aspects of a patient’s presentation, including past reactions to continuous observation, and the potential benefits and risks of observation for their overall recovery
Interviewees emphasised the importance of communication and collaboration between staff and patients	Communicate sensitively and empathically with patients to explain what observation entails Discuss the reasoning behind any decisions Acknowledge the difficulties of being observed Build mutual trust to enable agreement on taking positive risks within observation Take patients’ views about being observed and about their level of risk into account where possible, whilst acknowledging that some patients may seek to hide their level of risk or lack capacity to weigh up the pros and cons of observation whilst they are acutely ill
Interviewees emphasised the importance of a supportive and cohesive staff team	Involve all team members in reaching an agreement about patients’ level of observation Present a united front when communicating team decisions to patients and do not communicate any disagreements between staff Hand over information gained from observation to the rest of the team Encourage each-other to take positive risks and avoid a culture of blame Encourage staff to discuss difficult experiences with the team, in managerial supervision sessions, and during reflective practice Ensure that there are sufficient staff on the ward for observation shifts to be frequently rotated, and for adequate attention to be given to other patients

## Conclusions

Best practice in decision-making about continuous observation may be facilitated by making decisions in collaboration with patients where possible, and by staff supporting each-other in positive risk-taking. To achieve truly patient-centred decision-making, decisions about observation should not be influenced by staff’s own stress levels. To address the negative impact of staff stress on decision-making, it may be helpful to improve staff training, education and support structures.

## Electronic supplementary material

Below is the link to the electronic supplementary material.

Supplementary material 1 (DOCX 25 KB)
