# Supplementary material for: Staff and patient experiences of decision-making about continuous observation in psychiatric hospitals
Source: Soc Psychiatry Psychiatr Epidemiol. 2017 Feb 4;52(4):473–83. doi: 10.1007/s00127-017-1338-4 (PMC5380690; doi:10.1007/s00127-017-1338-4)
Supplement: Supplementary file 1 — Supplementary material 1 (DOCX 25 KB) [file 127_2017_1338_MOESM1_ESM.docx]

**Staff and patient experiences of decision-making about continuous observation in psychiatric hospitals**

Kirsten Barnicot, PhD.,^a,e^  Bryony Insua-Summerhayes, BSc.,^b^ Emily Plummer, MBBS,^a^ Alice Hart.,^c^ Chris Barker, PhD.,^b*^ and Stefan Priebe, FRC Psych.^d*^

* contributed equally

^a^ Centre for Mental Health, Department of Medicine, Imperial College London, Commonwealth Building, Du Cane Road, London, United Kingdom, W12 0NN.

^b^ Clinical, Educational and Health Psychology, Department of Medicine, University College London, 1-19 Torrington Place, London, United Kingdom , WC1E 7HB.

^c^  Department of Neuroscience, Physiology and Pharmacology, University College London, Gower Street, London, United Kingdom , WC1E 6BT.

^d^ Unit for Social and Community Psychiatry, Department of Medicine, Queen Mary University of London, Glen Road, London, United Kingdom , E13 8SP.

^e^ Corresponding author. Dr Kirsten Barnicot, Centre for Mental Health, Department of Medicine, Imperial College London, Commonwealth Buidling, Du Cane Road, London, United Kingdom , W12 0NN. Email: [k.barnicot@imperial.ac.uk](mailto:k.barnicot@imperial.ac.uk). Tel. +44 20 8383 4161. Fax. +44 20 8383 4160.

**Supplementary Table 1 – Additional Supporting Participating Quotes**

|  | **Supplementary Participant Quotes** |
| --- | --- |
| **Sub-theme 1.1**  **Invading privacy** | P02: “I tried closing the door many a time when I was having a shower but the door was just opened. I felt degraded.”  P04: “You can’t freely do what you want. So it became frustrating..... And having them follow you everywhere....At first you feel a bit bombarded by it”  P06: “When I was exhausted and just wanted to sleep, and a member of staff was stood outside the door and they were talking quite a lot with other members  of staff. …that was intrusive.. I felt very much the patient… I was pretty helpless at that point, you know, pretty feeble.”  P10: I feel like I don’t have freedom, like I can’t go anywhere, like all  the time escorted....like I am in jail now, in prison”  P11:“It’s like you’ve done something wrong, even though you know yourself that you’ve done nothing wrong. And they’re just looking at you… it’s like you’re in prison”  P25: “Degrading. Joke....I cannot comprehend why for the life of me for a woman [my age],you need to go to toilet with them?”  P27: “I felt like I was, like an animal at times. Being observed. I couldn’t do anything.”  S23: I felt like I was invading her personal space in a way, and I wanted to give her privacy, but at the same time I couldn’t, ‘cause her safety was paramount.”  S28: “They think that it's a breach of privacy...they feel restricted.... controlled. I think that's how I would feel if I was on the other side of the fence.” |
| **Sub-theme 1.2**  **Providing safety** | P01: “Obviously I needed it because I was in danger of hurting or self-harming myself… [it was] like a comfort blanket.”  P07:” I knew that they was looking out for me, so I wasn’t offended that perhaps my personal space was being invaded”  P10: “One thing is good. When the staff all the time 1 to 1 with me, I can control myself. I don’t do any silly things like hurting myself….. Because all the time someone watching me.”  P13: “I felt safe because I couldn’t trust myself, and I felt they were keeping me safe …it saved me from myself.”  P15: “At the time I was suicidal and someone was trying to stop me. It’s frustrating because you don’t understand.... you think ‘Why do you leave me to suffer?’”  P21: “It made me feel safer, because my self-harm felt very out of control... A bit of me wanted to self-harm and bit didn’t want to self-harm. It was nice to have the conflict helped a bit… I didn’t have the choice anymore.”  S09: “I had a few experiences where the patients were very grateful to the staff because they felt that at this point, nothing could actually help them and stop them feeling suicidal and prevent any suicide attempts.”  S16: “It does help the patient because sometime fear can lead them to feel  that they want to die; because they feel like they’re not safe on the ward, they feel intimidated by other patients. So when they feel that there’s a nurse with them, every minute, every hour, they feel safe.”  S25: “The benefits? We can save their lives and I mean that”.  S28: “It manages the risks, it limits the risks, it does work, evidence shows that”. |
| **Sub theme 2.1 A damaging intervention** | P10: “I feel like more depression when I been one to one…..I feel like that’s prison, so that’s why I feel more depression, more stress.”  P15: “There are times when it increases your distress level because there’s a person who gets on your nerves and then you get put with them. You’re ready to pull your fucking hair out.”  P23: One to ones, they, they’re just on your back, they piss me off.... You shut me in here I get very angry. …Following me around like a hawk!...Couldn’t even relax”  P27: “I just wanted to punch someone in the face. Like ‘what’s going on with you, why are you coming in here for? I’m in the bathroom!’”  P28: “I remember a member of staff getting really angry at me and it just made me worse because then I was stuck with someone who was angry with me for the next 2 hours… that made me a lot more distressed because I couldn’t get away from it”  S07: “During the one to one observations there’s social interaction, whereas when the observation is reduced there may be more isolation ….There almost becomes a dependency on the one to one observation ….the process may even be reinforcing self-harming behaviours.”  S08: “It can lead to sort of verbal and even physical aggression and hostility because of that perceived invasion of their privacy and personal space.”  S15: “They need to start taking responsibility for their own actions and being on a one-to-one is not necessarily going to enforce that. Is that going to help them at all, is that not taking the responsibility away from them?”  S15: “It made her more impulsive and she wanted to self-harm more because she had someone sitting there staring at her all the time.”  S17: “When somebody feels they’re being pressed by the system or there’s a conspiracy against them or they’re being watched all the time, it’s obviously very easy to see the observations as part of that conspiracy” |
| **Sub-theme 2.2 A short-term solution within a positive risk-taking framework** | S01: “I find sometimes I’m in a position where I’m on one to ones with someone who’s extremely aggressive.... Whereas actually if you just give them some space, keep them on 15 minute obs, they’ll cool down.”  S02: “ If you don’t know somebody, or they’re very high risk as in imminent, [then] ...acute crisis stage it makes perfect sense ... If someone’s got these ongoing ideas that are always there, then it’s – you start to question how effective it is. Whether it’s really making any difference at all.”  S04: “You have to review it on a daily basis.”  S05: “Sometimes obviously you have to compromise the privacy of the patient, but sometimes you have to take positive risks... this positive risk taking will lead you into deciding if someone can be taken off one to one*.”*  S12: “It’s a useful acute risk management tool....If they’re self-harming in a very serious way that’s likely to lead to permanent damage, as a short-term measure, in an acute situation, you might use one to one... if it’s something that’s a long-term problem and you need to be looking at long-term solutions....Because at some point you have to have positive risk taking.”  S25: “They self-harmed but a team decision had been made that they needed to be on intermittent because the one-to-one was actually making them worse…. in the end it turned out that it was the right decision to make”  S27: “It would be a last resort we would use on the ward, and it would be because the risk to self or others is so high that we have no other choice....we try to keep it to the minimum length of time” |
| **Sub theme 3.1 Decisions made without knowing or involving the patient** | P02: “When they knew my life story, I’ve been purifying for so long and still being put on one to one… I didn’t see the ethic in it. So I was very annoyed.”  P05: “Pencils and pens and stuff like that I can’t have in case I stab myself with them. It was unfair because ...the only thing I wanted to do was draw, and they took them off me.”  P12: “No-one really told me why I was on it….They didn’t tell me why they took me off it.”  P27: “I was still on observation for some reason. I started from scratch again. That’s how I felt”.  P28: “Quite often I’d be told ‘You’re coming off one to one today’, and then another member of staff would come and take over and I’d be back on one to one, and no one would’ve told me that I’d changed obs again.”  S02: “Sometimes people are put on one to one, when they’ve not been discussed sufficiently.”  S09: “Most of them, they understand when their risk lessens. ... I think it can be quite frustrating when the staff doesn’t listen or doesn’t understand or doesn’t want to see the change in presentation.”  S28: “One of the main challenges is if you don't know your patient… if it's the first time you've met the patient, you may not be too flexible with the privacy..”  S30: “What strikes me, being a consultant, is that I now have to act on, much more quickly, far less information. When I was a junior, we’d spend hours thinking about patients. But now it’s got to be bang bang bang. So it’s not ideal.” |
| **Sub-theme 3.2**  **A collaborative and individualised approach** | P13: “If I wanted privacy they gave me privacy... I’d go to the shower and leave the door open a little bit.....they’d just shout through and be like ‘Are you okay?’ and I’d say ‘Yeah I’m just in the shower, I’ll be out in five minutes.’”  P21: “I was involved in the choice to end one-to-one as I said I could cope without it. I prompted the decision.” P22: ”It was about my general safety and wellbeing....I knew that, I was well aware of that...they gave me a sheet, laying out why I’d been on observation and what kind of level of observation I was on.” P28: “If I was showering, the most helpful times were where the staff member just acted normally about it and said things like ‘Do you want me to shut the shower curtain and pass you stuff?’ rather than it being a case of the door has got to be wide open, and everyone can see in”. S02: “I do try to allow them privacy. If they want to shower, close the door, but understand that I will be knocking every so often to make sure you’re okay. And if you’re not going to respond, then I’ll open the door.” S07: “I would like to see a discussion being had between the clinician and the patient about the decision being made around observations... doing it in agreement with the patient, having them central in the decision making.” S15: “Explain to the patient why we’re doing this - it’s because actually we do care and we want to keep you safe, and if this behaviour reduces then we’re going to reduce our observations.” S27: “You're saying to the patient ‘I'm going to be with you for a while, what would you like to do for that one hour?’... That gives them the ownership.... autonomy.” |
| **Sub-theme 4.1**  **A stressed and fractured workforce** | P02: “Some of the nurses refused to do one to one.... refused to sit on the other  side of the door. And when she was outside the door she was constantly on the phone. And to me they didn’t care. They didn’t care if I purified every drop of my blood.... They said ‘I don’t know why you’re on one to ones. If you want to purify your blood you should be allowed to purify your blood!’”  P10: “Someone watching me - but when I went down and outside, then I ran away.... I just ran away from the hospital and I took overdose”.  P15: “Sometimes you wake up to find that there’s no one there because they think ‘Oh she’s gone to sleep’. So if you’re desperate then you’ll take that opportunity [to] harm yourself.”  P22: “There’s a nurse there sometimes, but sometimes they might leave their post to go and use the lavatory themselves or might be called to another thing going on in the ward.”  P28: “They said what a position I’d put them in because they’d made the decision to take me off one to one and within a couple of hours I was in A&E .... I was told that it was quite difficult for staff because there was quite a split in the team’s decision on whether I should be on one to one or not after that.”  S01: “I don’t think it’s right to be on one to one with someone who’s highly  aggressive, because I think staff are putting themselves at risk... I just don’t think it’s fair for me and other staff.”  S02: “The ones with people who are, the one to ones with people who are either very psychotic or very manic… those are mentally draining..... Because they are everywhere and anywhere at any time”.  S04: “. If you have four clients on the ward on one to one and you’re running the ward on full staff you need more members on site.... If you have agency staff who have never worked here, you have to spend time getting them to know what’s going on, how this is done, do they have relevant training... there you see you have a bit of a problem”.  S05: “We had quite a few situations last year where people made allegations, like  male versus female, female versus male, on both sides. And to safeguard both sides, I would not be able to sit in that room with a locked door. ...So that is where we unfortunately have to compromise in terms of the privacy is breached”.  S06: It’s prescribed when there are considerable risks. And that is something that is more tension laden… you are exposed to aggression... it also comes at a cost in terms of your resources being taken away and channelled only for that situation.”  S07: “As soon as you have someone on one to one observation that’s somebody out of your numbers being solely with that patient and then you’re thinking how much time have the nursing staff have got for the other patients ….. you need to start booking additional staff as well and that’s an additional drain on resources and adds to the cost pressure.” S15: “If there’s a staff shortage you end up having to do longer periods on the one-to-one ... it can get quite draining.” S21: “When anything goes wrong, you are the first person to give a full account of a person and therefore your observation skills should be a hundred percent, if somebody’s highly suicidal whereby a slip of an eye can cause something then you need to be alert!” S24: “He still managed to attack somebody, and that was quite difficult to accept the fact that I had failed.” |
| **Sub-theme 4.2**  **A team approach** | S04: “I’ve told staff if they have any issues, there’s charge nurses, there’s managers, there’s matrons… come and talk to us. And usually with supervision we pick this up. The charge nurse will sit with them and everyone discusses complex cases. So we talk about how much support we can provide or alternative methods we can use with abusive clients.’”  S13: “It’d be a case of the team making that decision and something would have to happen to warrant it.”  S14: “We’d do a general observation interacting with them, finding how things are going. We could comment to the doctors, the MDT team that this patient should go on one to one. And then followed by a risk assessment followed by the care plan. So we know exactly the reason why he’s going on one to one and that risk has been passed on to the whole team.”  S15: “My manager’s brilliant; she’s got this open door policy, so you can literally just walk into her office and just sit down, have a rant … for example, we had a patient on a one-to-one … it was a week of just constant abuse and that was just getting quite annoying.”  S18: “They are very supportive. If anything happens, if they find a patient being very aggressive they will come and support.”  S21: “If it gets too much we’ll swap with each other, because we’ve got a good team so when someone is struggling, then someone will come and help you… then we go to staff support meeting, and... you can all support each other.”  S26: “When it comes to taking them off, I normally encourage it to be an MDT thing, rather than to leave it to the nursing team only, because we have to share risks. At times we might think there is no risk here, but if you involve other disciplines, they might see the risk.”  S29: “In reflective practice, if you discuss with colleagues their experience of one to one, it kind of gives you an idea of how other staff are dealing with the problems and which one is actually working and which one is not working.” |
